# Survival after Acute Hemodialysis in Pennsylvania, 2005–2007: A Retrospective Cohort Study

**DOI:** 10.1371/journal.pone.0105083

**Published:** 2014-08-20

**Authors:** Sarah J. Ramer, Elan D. Cohen, Chung-Chou H. Chang, Mark L. Unruh, Amber E. Barnato

**Affiliations:** 1 Rutgers New Jersey Medical School, Newark, New Jersey, United States of America; 2 Division of General Internal Medicine, Department of Medicine, University of Pittsburgh School of Medicine, Pittsburgh, Pennsylvania, United States of America; 3 Department of Biostatistics, University of Pittsburgh Graduate School of Public Health, Pittsburgh, Pennsylvania, United States of America; 4 Division of Nephrology, Department of Internal Medicine, University of New Mexico School of Medicine, Albuquerque, New Mexico, United States of America; 5 Department of Clinical and Translational Science, University of Pittsburgh School of Medicine, Pittsburgh, Pennsylvania, United States of America; 6 Department of Health Policy and Management, University of Pittsburgh Graduate School of Public Health, Pittsburgh, Pennsylvania, United States of America; 7 The CRISMA Laboratory (Clinical Research, Investigation, and Systems Modeling of Acute Illness), Department of Critical Care Medicine, University of Pittsburgh, Pittsburgh, Pennsylvania, United States of America; National Cancer Institute, United States of America

## Abstract

**Background:**

Little is known about acute hemodialysis in the US. Here we describe predictors of receipt of acute hemodialysis in one state and estimate the marginal impact of acute hemodialysis on survival after accounting for confounding due to illness severity.

**Materials and Methods:**

This is a retrospective cohort study of acute-care hospitalizations in Pennsylvania from October 2005 to December 2007 using data from the Pennsylvania Health Care Cost Containment Council. Exposure variable is acute hemodialysis; dependent variable is survival following acute hemodialysis. We used multivariable logistic regression to determine propensity to receive acute hemodialysis and then, for a Cox proportional hazards model, matched acute hemodialysis and non-acute hemodialysis patients 1∶5 on this propensity.

**Results:**

In 2,131,248 admissions of adults without end-stage renal disease, there were 6,657 instances of acute hemodialysis. In analyses adjusted for predicted probability of death upon admission plus other covariates and stratified on age, being male, black, and insured were independent predictors of receipt of acute hemodialysis. One-year post-admission mortality was 43% for those receiving acute hemodialysis, compared to 13% among those not receiving acute hemodialysis. After matching on propensity to receive acute hemodialysis and adjusting for predicted probability of death upon admission, patients who received acute hemodialysis had a higher risk of death than patients who did not over at least 1 year of follow-up (hazard ratio 1·82, 95% confidence interval 1·68–1·97).

**Conclusions:**

In a populous US state, receipt of acute hemodialysis varied by age, sex, race, and insurance status even after adjustment for illness severity. In a comparison of patients with similar propensity to receive acute hemodialysis, those who did receive it were less likely to survive than those who did not. These findings raise questions about reasons for lack of benefit.

## Introduction

Acute hemodialysis (HD), defined as intermittent or continuous, non-peritoneal renal replacement therapy for patients without end-stage renal disease (ESRD), typically in the context of critical illness, is thought to save lives. Overall, however, the prognosis for patients requiring acute HD is poor, with estimates of in-hospital mortality in the literature ranging from 33% [1]-61% [2]. In a large high-quality clinical trial comparing different intensities of renal support for patients with acute kidney injury (AKI), 60-day mortality was nearly 53% [3]; in another such trial, 90-day mortality approached 45% [4]. Moreover, survivors are at high risk for continued renal failure [5]–[9] and decreased quality of life [10]–[12], and acute HD adds to the already substantial cost of care for the critically ill in the US [13], [14].

Although the US Renal Data System provides detailed information about patients with ESRD undergoing chronic HD, less is known about the epidemiology of acute HD. Several studies, most notably that of Xue and colleagues [1], have reported on the incidence and mortality of AKI among various populations of hospitalized patients in the US [2], [6], [8]. To our knowledge, however, no study has specifically focused on the predictors of or survival after acute HD.

In the current study, we describe predictors of the receipt of acute HD in a dataset encompassing over 2 million hospital admissions. We then attempt to estimate the marginal impact of acute HD on patient survival after accounting for confounding due to illness severity.

## Materials and Methods

### Data source

We conducted a retrospective cohort study of adult acute-care hospitalizations in Pennsylvania from October 2005 to December 2007 using data from the Pennsylvania (PA) Health Care Cost Containment Council (PHC4) linked to PA Department of Health death data through December 2008. PHC4 is an independent state agency that collects this data under mandate from the state government. The PHC4 data includes patient demographics, International Classification of Diseases, 9^th^ Revision, Clinical Modification (ICD-9-CM) diagnosis and procedure codes, and the predicted probability of inpatient death at the time of admission for all patients with certain pre-specified diagnosis-related groups (DRGs) and ICD-9-CM codes. (See Text S1. Diagnosis-related groups or International Classification of Diseases, 9^th^ Revision, Clinical Modification principal diagnoses for which the Pennsylvania Health Care Cost Containment Council required predicted probability of inpatient death to be calculated, by year.) The predicted probability of death (PPD) is based upon MediQual mortality models using key clinical findings abstracted from the chart during the first 48 hours of admission. These models significantly out-perform those based upon administrative fields alone, with average c-statistics of 0.86 [15]. It is important to note that the PHC4 data does not include any Current Procedural Terminology (CPT) codes; therefore, it is not possible to distinguish between intermittent HD and all other forms of renal replacement therapy in the PHC4 data.

### Exposure variable

**Figure 1:**
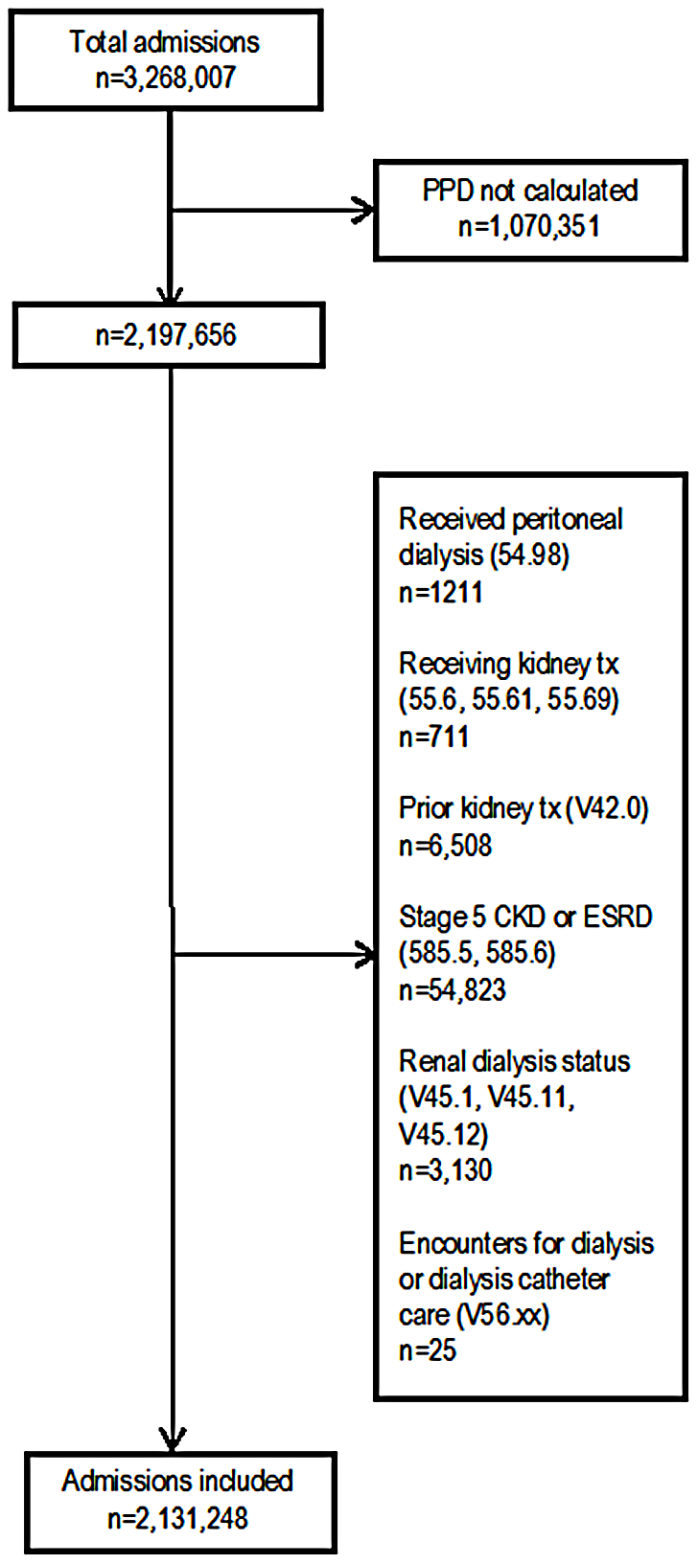
Sample selection process. PPD - predicted probability of death in-hospital, calculated from key clinical findings at admission; tx - transplant; CKD - chronic kidney disease; ESRD - end-stage renal disease.

Our principal exposure variable was acute HD. We defined acute HD as the receipt of HD (ICD-9-CM procedure code 39.95) by patients without ESRD. We excluded from all analyses admissions with ICD-9-CM diagnosis codes that would indicate current ESRD (585.6, V45.1, V45.11, V45.12, V56.xx), imminent ESRD, i.e., stage 5 chronic kidney disease (CKD) (585.5), or prior ESRD treated with kidney transplant (V42.0). We further excluded admissions with ICD-9-CM procedure codes for kidney transplant (55.6, 55.61, 55.69) or peritoneal dialysis (54.98), as these are procedures that only patients with ESRD undergo. For a flowchart depicting the sample selection process, see Figure 1.

### Dependent variable

The primary dependent variable is time to death, in days, from hospital admission. The minimum length of follow-up was one year.

### Covariates

Patient-level covariates included demographics, insurance status (commercial and/or Medicare, Medicaid with or without Medicare, and uninsured), principal diagnoses (classified into groups from ICD-9-CM codes using Agency for Healthcare Research and Quality Clinical Classification Software), and illness severity, as measured by the MediQual PPD (available for the 67% of admissions with one of the DRGs or ICD-9-CM codes for which PHC4 required that it be calculated). Hospital-level covariates included number of hospital beds, resident-to-bed ratio (a measure of teaching intensity), percent of patients who are black (a measure of racial concentration, correlated with treatment intensity and risk-adjusted mortality), and a Bayesian shrinkage estimator of the observed-to-expected ratio, i.e., standardized rate, of HD among patients at 95^th^ percentile of PPD upon admission (“end-of-life” HD treatment intensity) [16]. (See Figure S1. Standardized [observed-to-expected] hemodialysis use for patients at high probability of dying upon admission, by hospital, Pennsylvania 2001–2007.)

### Statistical analyses

We first descriptively summarized the characteristics of admissions in which patients received acute HD and compared them to admissions in which patients did not receive acute HD by employing chi-squared and t-tests. Using multivariable logistic regression with a random effect for hospital, we calculated a propensity score to receive acute HD based on patient- and hospital-level covariates for every admission, stratified by age (less than 65 years old and 65 years or older). We made the decision to stratify after a graphical analysis showed that the proportion of admissions with acute HD peaked at age 65 and declined thereafter.

On the basis of these propensity scores, we matched admissions with acute HD to those without acute HD in a ratio of 1∶5. If a patient had more than one admission, we used only the first admission, even if acute HD was not given in the first admission but was given in a subsequent admission. The rationale for this was to avoid bias, as a patient who underwent acute HD on a 2^nd^, 3^rd^, or subsequent admission and then died would seem to have a shorter survival if there was “resetting of the survival clock,” so to speak, to the time of that 2^nd^, 3^rd^, or subsequent admission. In order to improve the closeness of the matches, we employed matching with replacement, meaning that a non-HD admission could be re-used for matching with an HD admission. To gauge the effectiveness of matching, we used inverse probability treatment weighting to check the covariate balance between the acute HD and non-acute HD groups after propensity score matching. This method allowed us to compare pre- and post-matching samples more accurately since it maintained the original sample size. (See Figure S2. Covariate balance before and after inverse probability treatment weighting by propensity score.)

We plotted Kaplan-Meier survival curves of time to death, up to one year from admission, for admissions with and without acute HD. We then used Cox proportional hazards models to assess the effect of acute HD on survival, with covariate adjustment. Covariates included only patient-level variables, as hospital-level variables were not significantly associated with survival. To reduce indication (severity) bias, we calculated hazards of dying among admissions with and without acute HD, matched for propensity to receive acute HD, with covariate adjustment. We accounted for dependence between patients within a hospital by using the Huber-White sandwich estimator for standard errors. We performed all analyses with Stata version 11 (StataCorp, College Station, TX).

### Sensitivity analyses

Along with our main analyses, we performed 3 sensitivity analyses. The first only involved the calculation of propensity scores. Due to the possibility that our sample selection process might have incorrectly classified some HD given to ESRD patients as acute HD, we dropped all patients with Medicare, given that ESRD is a Medicare-qualifying condition, as well as all patients 65 years or older, given that most of them have Medicare, and again ran the multivariable logistic regression models.

The next two sensitivity analyses involved both the calculation of propensity scores and the survival modeling. One used the sample selection process depicted in Figure 1 but then further limited the sample to only admissions that included a stay in the intensive care unit (ICU). We then conducted the same statistical procedures as in the main analysis.

The final sensitivity analysis used the sample selection process depicted in Figure 1 but did not exclude admissions for which the MediQual PPD was not calculated. Instead of using the MediQual PPD as the main risk adjustor, we used the Charlson Comorbidity Index [17], [18]. Once again, we then carried out the same statistical procedures as in the main analysis.

### Ethics statement

We conducted this research under a data use agreement with PHC4, which acted as an honest broker to merge hospital discharge data with PA Department of Health death records. The University of Pittsburgh Institutional Review Board reviewed and approved the study, which met US federal criteria for exemption from written informed consent because data were not connected to any patient identifiers.

## Results

The characteristics of the full sample by acute HD status are detailed in Table 1. Patients who received acute HD differed significantly from those who did not in age, sex, race, and insurance status. Men (P<0.001), black patients (P<0.001), and insured patients (P<0.001) were all more likely to receive acute HD. The most common principal diagnoses among those who received acute HD were acute and unspecified renal failure; septicemia; non-hypertensive congestive heart failure; respiratory failure, insufficiency, or arrest; and diabetes mellitus with complications. As would be expected, acute HD patients were sicker, as reflected in their higher PPD on admission (P<0.001) and higher mortality at every time point (P<0.001 for all).

**Table 1:** Characteristics of full and matched samples by acute hemodialysis status, Pennsylvania 2005–2007.

	Full sample			Matched sample		
	No acute HD	Acute HD	P	No acute HD	Acute HD	P
Admissions^a^ (n)	2,124,591	6,657		17,415	3,483	
Patients (n)	1,283,053	6,113		16,167	3,483	
Female (%)	1,201,352 (57)	3,043 (46)	<0.001	7,856 (45)	1,555 (45)	0.615
Age (yrs)	65.0±17.9	65.5±15.0	0.024	65.2±15.9	64.4±15.5	0.004
Age (%)^b^			<0.001			0.026
21–49	452,764 (21)	1,034 (16)		2,982 (17)	626 (18)	
50–59	321,349 (15)	1,179 (18)		2,883 (17)	625 (18)	
60–69	358,797 (17)	1,439 (22)		3,863 (22)	746 (21)	
70–79	454,958 (21)	1,697 (25)		4,174 (24)	851 (24)	
80+	536,723 (25)	1,308 (20)		3,513 (20)	635 (18)	
Race (%)^b^			<0.001			<0.001
White	1,792,633 (84)	4,899 (74)		13,065 (75)	2,463 (71)	
Black	218,565 (10)	1,393 (21)		3,496 (20)	816 (23)	
Hispanic	38,395 (1.8)	141 (2.1)		292 (1.7)	80 (2.3)	
Asian/Pacific Islander	7,798 (0.37)	27 (0.41)		84 (0.48)	20 (0.57)	
Other/unknown	67,200 (3.2)	197 (3)		478 (2.7)	104 (3)	
Insurance (%)^b^			<0.001			<0.001
Commercial and/or Medicare	1,688,748 (80)	4,642 (70)		12,646 (73)	2,418 (69)	
Medicaid with/without Medicare	404,928 (19)	1,969 (30)		4,601 (26)	1,037 (30)	
Uninsured	30,142 (1.4)	44 (0.66)		168 (0.96)	28 (0.8)	
Primary diagnosis (%)						
Acute and unspecified renal failure	37,021 (1.7)	1,659 (25)	<0.001	4,087 (23)	816 (23)	0.959
Septicemia (except in labor)	55,833 (2.6)	900 (14)	<0.001	2,041 (12)	460 (13)	0.014
Congestive heart failure, non-hypertensive	121,786 (5.7)	547 (8.2)	<0.001	1,204 (6.9)	234 (6.7)	0.678
Respiratory failure, insufficiency, or arrest	33,132 (1.6)	328 (4.9)	<0.001	820 (4.7)	173 (5)	0.513
Diabetes mellitus with complications	40,435 (1.9)	323 (4.9)	<0.001	912 (5.2)	176 (5.1)	0.656
Predicted probability of death upon admission^c^	0.031±0.084	0.14±0.2	<0.001	0.11±0.21	0.13±0.19	0.002
Mortality (%)						
During admission	50,478 (2.4)	1,258 (19)	<0.001	1,480 (8.5)	589 (17)	<0.001
By 90 d post-admit date	194,257 (9.1)	2,309 (35)	<0.001	3,047 (17)	1,059 (30)	<0.001
By 1 yr post-admit date	271,519 (13)	2,837 (43)	<0.001	3,793 (22)	1,315 (38)	<0.001
By 90 d post-admit date, conditional on surviving to discharge	143,873 (6.9)	1,061 (20)	<0.001	1,571 (9.9)	474 (16)	<0.001
By 1 yr post-admit date, conditional on surviving to discharge	221,041 (11)	1,579 (29)	<0.001	2,313 (15)	726 (25)	<0.001

Continuous variables expressed as mean ± standard deviation; categorical variables expressed as n (%).

T-tests used for continuous variables; chi-squared tests used for categorical variables.

a All means and proportions are based on admissions, not individual patients.

b Due to rounding, percentages may not add up to 100.

c Based upon proprietary MediQual mortality model using key clinical findings abstracted from the chart during the first 48 hours of admission.

As shown in Table 2, in multivariable logistic regression, age, sex, race, and PPD were independently associated with receipt of acute HD. For patients less than 65 years old, increasing age predicted higher odds of receiving acute HD (P = 0.001); for patients 65 years or older, increasing age predicted lower odds of receiving acute HD (P<0.001). In both age groups, male and black patients were more likely to receive acute HD (P<0.001 for each). In the under-65 age group, being uninsured was associated with lower odds of receiving acute HD (P<0.001). In both age groups, admission to a hospital with a larger bed count (P<0.001 in under-65, P = 0.005 in 65 and older, data not shown) and higher standardized rate of HD among patients at a high probability of dying upon admission were associated with receipt of acute HD (P<0.001 for both age groups, data not shown).

**Table 2:** Independent predictors of receipt of acute hemodialysis, Pennsylvania 2005–2007.^a^

	Age<65		Age≥65	
	Adjusted odds ratio	95% confidence interval	Adjusted odds ratio	95% confidence interval
Age (per year)	1.01	1.00–1.01	0.95	0.94–0.95
Female	0.79	0.73–0.86	0.85	0.79–0.91
Black	1.37	1.24–1.52	1.54	1.37–1.73
Uninsured (vs. Medicare/commercial)	0.49	0.35–0.68	0.58	0.27–1.25
Uninsured (vs. Medicaid ± Medicare)	0.35	0.25–0.48	0.58	0.27–1.27
Primary diagnoses (top 5 by prevalence over all ages)				
Acute and unspecified renal failure	57.9	51.4–65.2	31.3	28.2–34.6
Septicemia (except in labor)	10.8	9.35–12.6	5.57	4.89–6.36
Congestive heart failure, non-hypertensive	6.30	5.31–7.49	3.80	3.34–4.33
Respiratory failure, insufficiency, or arrest	5.55	4.54–6.77	3.12	2.63–3.71
Diabetes mellitus with complications	7.19	6.06–8.54	8.22	6.85–9.86
Predicted probability of inpatient death (per 1% increase)	49.6	40.0–61.4	39.0	32.9–46.3

a Based on the full sample.

The characteristics of the matched sample by acute HD status are displayed in Table 1. Of the 6113 patients who received acute HD in the time period covered by the study, only 3483 did so in their first admission and were therefore eligible for matching. After matching, there was no longer a significant difference between the groups in sex (P = 0.615). A significant difference persisted for age measured continuously (P = 0.004), but in the matched sample the patients who received acute HD actually had a lower mean age, as opposed to in the full sample, in which they had a higher mean age. Significant differences also persisted for race (P<0.001), insurance status (P<0.001), and PPD on admission (P = 0.002), but as Figure S2in the supplemental digital content demonstrates, these differences were very much attenuated in the matched sample. Patients who did and did not receive acute HD continued to differ significantly in mortality at every time point (P<0.001 for all).

**Figure 2:**
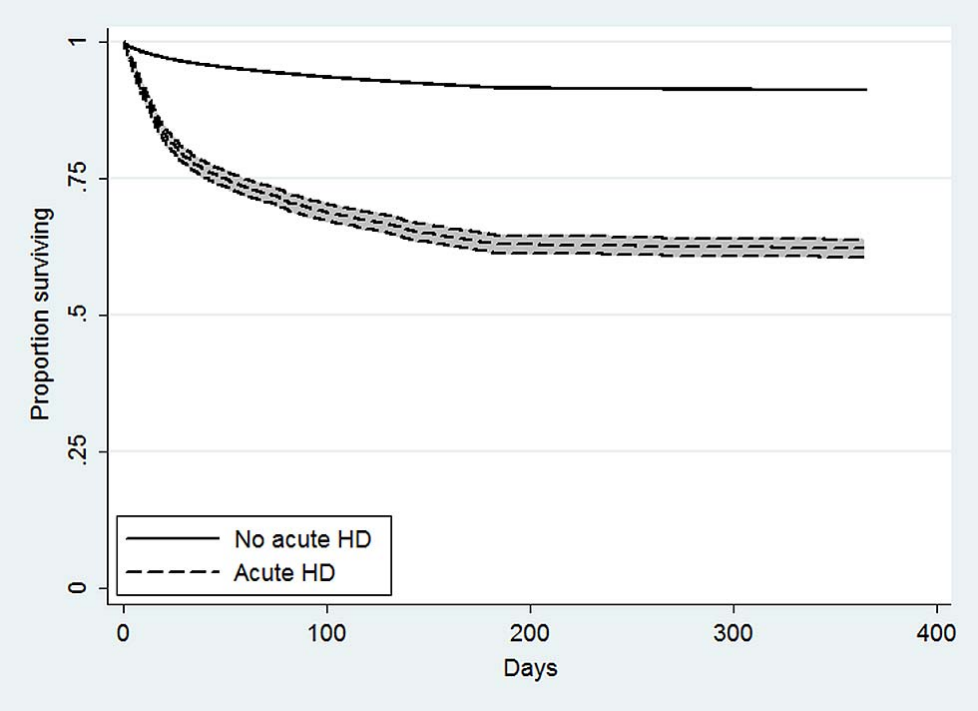
Unadjusted survival among patients with and without acute hemodialysis, Pennsylvania 2005–2007. Kaplan-Meier survival curves for patients who did and did not receive acute hemodialysis, with 95% confidence intervals.

**Figure 3:**
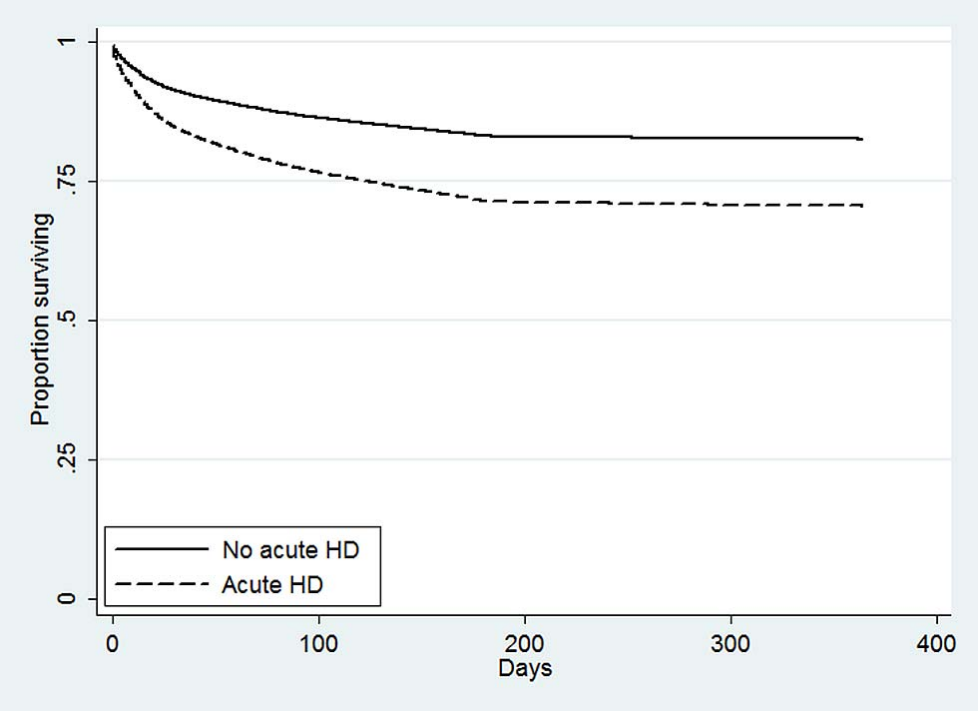
Adjusted survival among propensity-score matched patients with and without acute hemodialysis, Pennsylvania 2005–2007. Cox-adjusted survival curves with covariate adjustment for patients who did and did not receive acute hemodialysis after matching on propensity to receive acute hemodialysis. Variables included in covariate risk adjustment included age; female sex; black race; insurance with Medicare or private insurance vs. no insurance; insurance with Medicaid and/or Medicare vs. no insurance; MediQual predicted probability of death; and the top 25 Clinical Classification Software admission diagnoses for people who received acute hemodialysis, with the exception of hypertension with complications and secondary hypertension (#6) and peripheral and visceral atherosclerosis (#25), which were dropped in the model selection phase.

Figure 2 shows Kaplan-Meier curves for patients who did and did not receive acute HD. As would be expected based upon their illness severity, the patients who received acute HD were more likely to die throughout the follow-up period. In the fully adjusted Cox model before propensity score matching, the acute HD patients had a higher risk of death (hazard ratio [HR] 1.78, 95% confidence interval [CI] 1.58–2.00). The survival difference persisted essentially unchanged in the fully adjusted Cox model after propensity score matching (HR 1.82, 95% CI 1.68–1.97; Figure 3).

The sensitivity analysis excluding patients insured by Medicare or 65 years or older produced odds ratios qualitatively unchanged from those obtained from the main analysis. The sensitivity analysis restricted to patients admitted to the ICU differed notably from the main analysis in that, for patients younger than 65, increasing age was not significantly associated with higher odds of receiving acute HD (odds ratio 1.00, 95% CI 1.00–1.01, P = 0.85). In the fully adjusted Cox model after propensity score matching, however, the acute HD patients still had a higher risk of death (HR 1.77, 95% CI 1.64–1.92). In the sensitivity analysis that used the Charlson Comorbidity Index rather than the MediQual PPD as the means of risk adjustment, the sample size increased by almost 50% (2,131,248 to 3,174,283) since the admissions for which PPD was not calculated were included. In this larger sample, the hazard ratio for death after acute HD in the fully adjusted Cox model after propensity score matching increased (HR 2.60, 95% CI 2.41–2.80).

## Discussion

This work represents one of the first descriptions of the epidemiology of acute HD not limited to Medicare beneficiaries in a large US population. Receipt of acute HD varied by age, sex, race, and insurance status, even after adjustment for patients' underlying illness severity. Furthermore, in a comparison of patients with similar propensity to receive acute HD and adjusted for predicted probability of death upon admission, those who did receive acute HD had a higher risk of death for at least one year compared to those who did not.

Our findings that being male or black are independent predictors of receipt of acute HD are consistent with previous findings that these groups are more likely to receive other intensive interventions such as mechanical ventilation [19]–[21], pulmonary artery catheters [19], [20], and cardiopulmonary resuscitation [22]. Other authors have also previously reported a higher rate of renal replacement therapy among critically ill men [19]. Similarly, our finding that the uninsured are much less likely than those with any type of insurance to receive acute HD is in line with a recent report by Lyon and colleagues, who, also working with the PHC4 data, noted that uninsured patients less than 65 years old had a significantly lower probability of receiving acute HD, tracheostomies, and central venous catheters [23]. It should be noted, however, that Lyon's strategy for differentiating acute from chronic HD relied on older ICD-9-CM codes that didn't allow for separation of CKD and ESRD [24]. As such, their strategy may have unnecessarily eliminated from acute HD analyses some CKD patients whose HD was in fact acute. At first glance, after seeing this kind of disparity between the insured and uninsured, one might attribute it to healthcare providers' reluctance to offer expensive therapies to patients who are unlikely to ever be able to pay for them. As Lyon pointed out, though, the disparity could result more from choices by the uninsured patients and their surrogates than from healthcare providers' decisions—unconscious or otherwise—to forego these interventions in the uninsured. The reasons underlying the observed disparity merit further investigation, perhaps using survey-based or qualitative methods.

Perhaps the most striking finding from our study is the increased mortality risk for patients who received acute HD, even after robust risk-adjustment, including propensity score matching. Even after we limited the sample to the sickest patients, those admitted to the ICU, this increased mortality risk persisted. This finding runs counter to the commonly accepted notion that acute HD decreases mortality for those in whom it is initiated. Certainly, despite our best efforts to match by propensity to receive acute HD patients who did and did not receive it, the possibility of unmeasured confounding by illness severity remains. Though a principal diagnosis of AKI was a covariate in our propensity scoring and survival models, we could not match patients on severity of AKI (e.g., by serum BUN or creatinine or urine output), as our dataset did not include that information.

However, we also cannot rule out the possibility that acute HD is deleterious to patient survival. Elseviers and colleagues reported similar results from a nonrandomized prospective study of 1,303 patients in 9 ICUs [25]. They found that differences in illness severity did not account for the higher in-hospital mortality observed in patients who received acute HD. Intriguingly, they also noted wide variations between ICUs in initiation of acute HD, underscoring the general lack of consensus in nephrology and critical care about the relative indications for this therapy. In our study, we attempted to account for between-institution variations by including each hospital's standardized rate of HD among patients at a high probability of dying in our propensity score calculation and appropriately adjusting Cox model standard errors for clustering of patients within hospitals.

Two even more recent studies, though they did not find higher mortality with acute HD, did find lack of survival benefit. For Clec'h and colleagues, this lack of benefit was in patients who received acute HD versus those who did not after matching on propensity to receive acute HD using detailed clinical information [26]. For Wilson and colleagues, this lack of benefit was in patients who developed severe AKI on days other than Sundays compared to patients who developed severe AKI on Sundays, despite lower frequency of acute HD initiation on Sundays [27]. Moreover, Wilson and colleagues, in their most recent study, observed an association between dialysis for AKI and increased survival when dialysis was initiated in patients with higher serum creatinine but not when it was initiated in patients with lower serum creatinine. In fact, in patients with lower serum creatinine, initiation of dialysis for AKI was associated with increased mortality [28]. Though the reasons that acute HD might exert either no effect or a negative effect on patient survival are far from clear and merit further research, the known risks of HD—episodic hypotension, an indwelling catheter, immune and coagulation disturbances, changes in medication clearance, to name a few—all might play a role.

Our study has several strengths that should be noted. Our sample includes over 2 million observations from a dataset that captured admissions of all adults in almost every hospital in a populous US state, not just Medicare beneficiaries or hospitals within a particular commercial or government healthcare system. Our analyses benefitted from the precise risk adjustment of the MediQual model for predicted probability of death. Furthermore, by restricting our sample to admissions from the last quarter of 2005 onwards, we were able to take advantage of updated ICD-9-CM codes that allow for separation of CKD and ESRD. As such, we can have reasonable confidence in our ability to differentiate between acute and chronic HD using administrative data.

This study is not without weaknesses, however. Though we carefully crafted our strategy to exclude ESRD patients from the sample and backed it up with a sensitivity analysis, without medical records we are unable to fully validate the strategy. Despite adjusting for a wide variety of covariates in our models and propensity-score matching patients who received acute HD with patients who did not, as well as replicating our main results with a sensitivity analysis limited to patients sick enough to be admitted to the ICU, there is still likely to be some degree of unmeasured confounding contributing to the survival difference between the groups. In the most extreme case, if the only patients dialyzed were those who would have definitely died from their illness, then acute HD had to confer a survival benefit, as some patients did survive. While we cannot exclude this possibility using our dataset, we mitigated it to the fullest extent that our dataset would allow. Finally, in order to use the precise risk adjustment of the MediQual model, we excluded all those admissions for which PHC4 did not require calculation of predicted probability of death. Our sample must therefore be considered nonrandom. In fact one of our sensitivity analyses did demonstrate that the inclusion of all admissions in our sample would have altered the results. But the most important finding, the hazard ratio for death in patients who received acute HD versus those who did not, actually became larger in this case.

Taken in the context of previous research, our findings regarding predictors of acute HD suggest factors other than clinical indication at play in determining who receives this therapy; our findings regarding survival after acute HD raise questions about its presumed benefits. Given the extraordinary costs of intensive interventions such as acute HD in the US, and the ever-expanding population of patients who might become ill enough to be viewed as candidates for it, healthcare providers and policymakers alike must carefully consider why exactly patients receive the therapies they do and whether those reasons impact eventual outcomes.

## Supporting Information

Figure S1 **Standardized (observed-to-expected) hemodialysis use for patients at high probability of dying upon admission, by hospital, Pennsylvania 2001–2007.** Each dot represents 1 hospital.
(DOCX)

Figure S2 **Covariate balance before and after inverse probability treatment weighting by propensity score.** Movement of the open dots towards a Z-value of 0 represents better covariate balance after weighting, with a value of 0 reflecting perfect balance.
(DOCX)

Text S1 **Diagnosis-related groups or International Classification of Diseases, 9^th^ Revision, Clinical Modification principal diagnoses for which the Pennsylvania Health Care Cost Containment Council required predicted probability of inpatient death to be calculated, by year.**
(DOCX)
