# Supplementary material for: Survival after Acute Hemodialysis in Pennsylvania, 2005–2007: A Retrospective Cohort Study
Source: PLoS One. 2014 Aug 20;9(8):e105083. doi: 10.1371/journal.pone.0105083 (PMC4139312; doi:10.1371/journal.pone.0105083)

**Figure S1. Standardized (observed-to-expected) hemodialysis use for patients at high probability of dying (HPD) upon admission, by hospital, Pennsylvania 2001-2007.** Each dot represents 1 hospital.


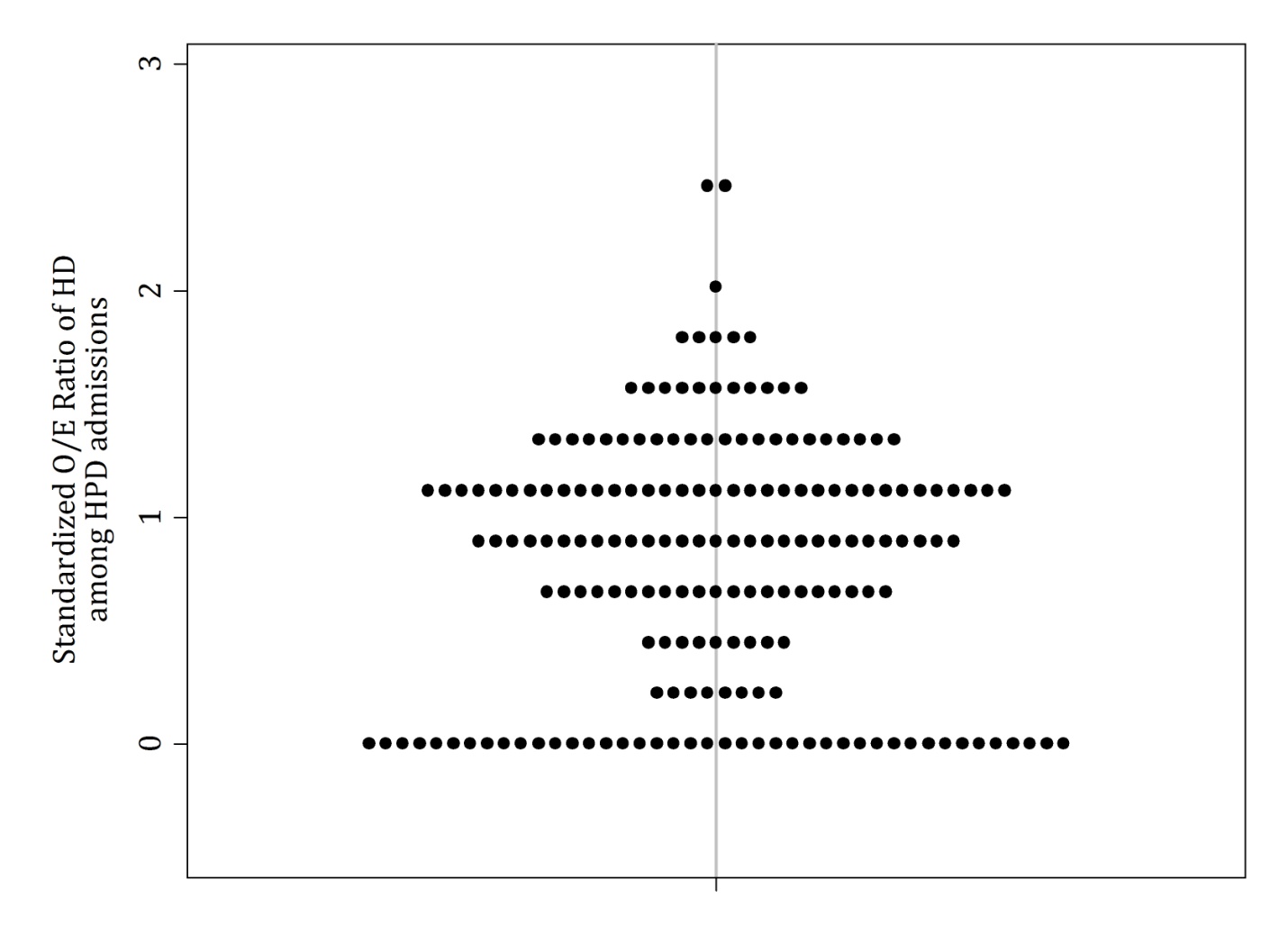

Supplement: Figure S1 — Standardized (observed-to-expected) hemodialysis use for patients at high probability of dying upon admission, by hospital, Pennsylvania 2001–2007. Each dot represents 1 hospital. (DOCX) [file pone.0105083.s001.docx]
