# Supplementary material for: Survival after Acute Hemodialysis in Pennsylvania, 2005–2007: A Retrospective Cohort Study
Source: PLoS One. 2014 Aug 20;9(8):e105083. doi: 10.1371/journal.pone.0105083 (PMC4139312; doi:10.1371/journal.pone.0105083)
Supplement: Figure S2 — Covariate balance before and after inverse probability treatment weighting by propensity score. Movement of the open dots towards a Z-value of 0 represents better covariate balance after weighting, with a value of 0 reflecting perfect balance. (DOCX) [file pone.0105083.s002.docx]

**Figure S2. Covariate balance before and after inverse probability treatment weighting by propensity score.** Movement of the open dots towards a Z-value of 0 represents better covariate balance after weighting, with a value of 0 reflecting perfect balance.

**
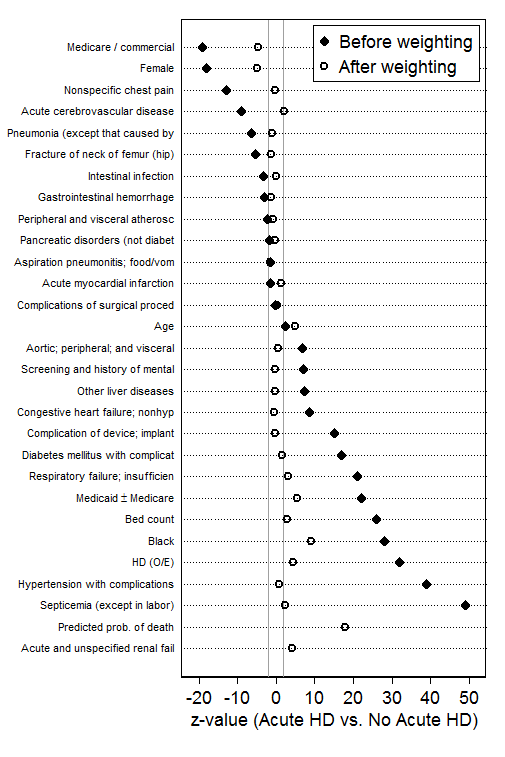
**
