# Supplementary material for: Survival after Acute Hemodialysis in Pennsylvania, 2005–2007: A Retrospective Cohort Study
Source: PLoS One. 2014 Aug 20;9(8):e105083. doi: 10.1371/journal.pone.0105083 (PMC4139312; doi:10.1371/journal.pone.0105083)
Supplement: Text S1 — Diagnosis-related groups or International Classification of Diseases, 9th Revision, Clinical Modification principal diagnoses for which the Pennsylvania Health Care Cost Containment Council required predicted probability of inpatient death to be calculated, by year. (DOCX) [file pone.0105083.s003.docx]

**Text S1.** Diagnosis-related groups or International Classification of Diseases, 9^th^ Revision, Clinical Modification principal diagnoses for which the Pennsylvania Health Care Cost Containment Council required predicted probability of inpatient death to be calculated, by year.

**2005-2**

***Heart Attack (DRGs)***

121 Circulatory disorders w AMI & major comp, discharged alive

122 Circulatory disorders w AMI & w/o major comp, discharged alive

123 Circulatory disorders w AMI, expired

***Heart Failure (DRGs)***

127 Heart failure & shock

***Chest Pain (DRGs)***

143 Chest pain

***Abnormal Heartbeat (DRGs)***

115 Permanent cardiac pacemaker implant w AMI, heart failure, or shock or AICD lead or generator proc

116 Other cardiac pacemaker inplantation

117 Cardiac pacemaker revision except device replacement

118 Cardiac pacemaker device replacement

124 Circulatory disorders except AMI, w card cath & complex diag

125 Circulatory disorders except AMI, w card cath w/o complex diag

138 Cardiac arrhythmia & conduction disorders w cc

139 Cardiac arrhythmia & conduction disorders w/o cc

***Coronary Bypass (DRGs)***

103 Heart Transplant or heart assist system

106 Coronary bypass w PTCA

107 Coronary bypass w cardiac cath

108 Other cardiothoracic procedures

109 Coronary bypass w/o cardiac cath

515 Cardiac defibrillator implant w/o cardiac cath

525 Other heart assist system implant

535 Cardiac defibrillator implant w cardiac cath w prin. Dx AMI, Heart Failure or Shock

536 Cardiac defibrillator implant w cardiac cath w/o prin. Dx AMI, Heart Failure or Shock

***Heart Value Replacement (DRGs)***

104 Cardiac valve proc & other major cardiothoracic proc w cardiac cath

105 Cardiac valve proc & other major cardiothoracic proc w/o cardiac cath

***Percutaneous Transluminal Coronary Angioplasty (DRGs)***

516 Percutaneous cardiovascular proc w AMI

517 Percutaneous cardiovascular proc w/o AMI, w coronary artery stent implant

518 Percutaneous cardiovascular proc w/o AMI, w/o coronary artery stent implant

526 Percutaneous cardiovascular proc w drug-eluting stent w AMI

527 Percutaneous cardiovascular proc w drug-eluting stent w/o AMI

***Pneumonia (DRGs)***

079 Respiratory infections & inflammations, age > 17 w cc

080 Respiratory infections & inflammations, age > 17 w/o cc

089 Simple pneumonia & pleurisy, age > 17 w cc

090 Simple pneumonia & pleurisy, age > 17 w/o cc

***Asthma (DRGs)***

088 Chronic obstructive pulmonary disease

096 Bronchitis & asthma, age > 17 w cc

097 Bronchitis & asthma, age > 17 w/o cc

098 Bronchitis & asthma, age 0-17

***Respiratory Failure (DRGs)***

087 Pulmonary edema and respiratory failure

***Blood Clot in Lung (DRGs)***

078 Pulmonary embolism

***Lung Repair (DRGs)***

075 Major chest procedures

076 Other respiratory system OR proc w cc

077 Other respiratory system OR proc w/o cc

***Hypotension (DRGs)***

141 Syncope and collapse w cc

142 Syncope and collapse w/o cc

***Blood Clot in Extremities (DRGs)***

128 Deep vein thrombophlebitis

130 Peripheral vascular disorders w cc

131 Peripheral vascular disorders w/o cc

***Vascular Repair (DRGs)***

110 Major cardiovascular procedures w cc

111 Major cardiovascular procedures w/o cc

478 Other vascular procedures w cc

479 Other vascular procedures w/o cc

***Stroke (DRGs)***

014 Intracranial hemorrhage or Cerebral Infarction

015 Nonspecific cerebrovascular & precerebral occlusion w/o infarction

***Removal of Blockage in Head & Neck Vessel (DRGs)***

533 Extracranial vascular procedures w CC

534 Extracranial vascular procedures w/o CC

***Craniotomy (DRGs)***

001 Craniotomy, age > 17 w cc

002 Craniotomy, age > 17 w/o cc

528 Intracranial vascular procedures w Prin. Dx of Hemorrhage

529 Ventricular shunt procedures w CC

530 Ventricular shunt procedures w/o CC

543 Craniotomy w/ implant of chemo agent or acute complex CNS principal diagnosis

***Diabetes (Principal Diagnosis)***

25000 DM2 UNCOMP NSU

25001 DM1 UNCOMP NSU

25002 DM2 UNCOMP UNC

25003 DM1 UNCOMP UNC

25010 DM2 W KETOACIDOSIS NSU

25011 DM1 W KETOACIDOSIS NSU

25012 DM2 W KETOACIDOSIS UNC

25013 DM1 W KETOACIDOSIS UNC

25020 DM2 W HYPEROSMOLARITY NSU

25021 DM1 W HYPEROSMOLARITY NSU

25022 DM2 W HYPEROSMOLARITY UNC

25023 DM1 W HYPEROSMOLARITY UNC

25030 DM2 W COMA NEC, NSU

25031 DM1 W COMA NEC, NSU

25032 DM2 W COMA NEC, UNC

25033 DM1 W COMA NEC, UNC

25040 DM2 W RENAL MANIFEST, NSU

25041 DM1 W RENAL MANIFEST, NSU

25042 DM2 W RENAL MANIFEST, UNC

25043 DM1 W RENAL MANIFEST, UNC

25050 DM2 W EYE MANIFEST, NSU

25051 DM1 W EYE MANIFEST, NSU

25052 DM2 W EYE MANIFEST, UNC

25053 DM1 W EYE MANIFEST, UNC

25060 DM2 W NEURO MANIFEST, NSU

25061 DM1 W NEURO MANIFEST, NSU

25062 DM2 W NEURO MANIFEST, UNC

25063 DM1 W NEURO MANIFEST, UNC

25070 DM2 W CIRC DISORD, NSU

25071 DM1 W CIRC DISORD, NSU

25072 DM2 W CIRC DISORD, UNC

25073 DM1 W CIRC DISORD, UNC

25080 DM2 W MANIFEST NEC, NSU

25081 DM1 W MANIFEST NEC, NSU

25082 DM2 W MANIFEST NEC, UNC

25083 DM1 W MANIFEST NEC, UNC

25090 DM2 Q COMP NOS, NSU

25091 DM1 Q COMP NOS, NSU

25092 DM2 Q COMP NOS, UNC

25093 DM1 Q COMP NOS, UNC

***Digestive Disease (DRGs)***

174 GI hemorrhage w cc

175 GI hemorrhage w/o cc

180 GI obstruction w cc

181 GI obstruction w/o cc

182 Esophagitis, gastroenteritis, and miscellaneous digestive disorders, age > 17 w cc

183 Esophagitis, gastroenteritis, and miscellaneous digestive disorders, age > 17 w/o cc

188 Other digestive system diagnoses, age > 17, w cc

189 Other digestive system diagnoses, age > 17, w/o cc

***Liver Disease (DRGs)***

202 Cirrhosis & alcoholic hepatitis

203 Malignancy of hepatobiliary system or pancreas

204 Disorders of pancreas except malignancy

205 Disorders of liver except malignancy, cirrhosis and alcoholic hepatitis w cc

***Colorectal Repair (DRGs)***

146 Rectal resection w cc

147 Rectal resection w/o cc

148 Major small & large bowel procedures w cc

149 Major small & large bowel procedures w/o cc

***Gallbladder Removal (DRGs)***

195 Cholecystectomy w common duct exploration w cc

196 Cholecystectomy w common duct exploration w/o cc

197 Cholecystectomy except by laparoscope w/o common duct exploration w cc

198 Cholecystectomy except by laparoscope w/o common duct exploration w/o cc

493 Laparoscopic cholecystectomy w/o common duct exploration w cc

494 Laparoscopic cholecystectomy w/o common duct exploration w/o cc

***Stomach & Small Intestine Repair (DRGs)***

154 Stomach, esophageal, and duodenal procedures, age > 17 w cc

155 Stomach, esophageal, and duodenal procedures, age > 17 w/o cc

***Kidney Failure (DRGs)***

316 Renal failure

***Kidney & Urinary Tract Infection (DRGs)***

320 Kidney & urinary tract infections, age > 17 w cc

321 Kidney & urinary tract infections, age > 17 w/o cc

***Prostatectomy (DRGs)***

306 Prostatectomy w cc

307 Prostatectomy w/o cc

334 Major male pelvic procedures w cc

335 Major male pelvic procedures w/o cc

336 Transurethral prostatectomy w cc

337 Transurethral prostatectomy w/o cc

***Medical Back (DRGs)***

243 Medical back problems

***Major Joint Repair (DRGs)***

209 Major joint & limb reattachment procedures of lower extremity

210 Hip & femur procedures except major joint proc, age > 17 w cc

211 Hip & femur procedures except major joint proc, age > 17 w/o cc

471 Bilateral or multiple major joint proc of lower extremity

***Neck/Back Repair (DRGs)***

496 Combined anterior/posterior spinal fusion

497 Spinal fusion except cervical w cc

498 Spinal fusion except cervical w/o cc

499 Back & neck procedures except spinal fusion w cc

500 Back & neck procedures except spinal fusion w/o cc

519 Cervical spinal fusion w cc

520 Cervical spinal fusion w/o cc

***Breast Cancer (DRGs)***

257 Total mastectomy for malignancy w cc

258 Total mastectomy for malignancy w/o cc

259 Subtotal mastectomy for malignancy w cc

260 Subtotal mastectomy for malignancy w/o cc

265 Skin graft &/or debridement except for skin ulcer or cellulites w cc

266 Skin graft &/or debridement except for skin ulcer or cellulites w/o cc

***Hysterectomy (DRGs)***

353 Pelvic evisceration, radical hysterectomy & radical vulvectomy

354 Uterine & adnexa proc for non-ovarian/adnexal malignancy w cc

355 Uterine & adnexa proc for non-ovarian/adnexal malignancy w/o cc

357 Uterine & adnexa proc for ovarian or adnexal malignancy

358 Uterine & adnexa proc for non-malignancy w cc

359 Uterine & adnexa proc for non-malignancy w/o cc

***Infectious Disease (DRGs)***

415 OR procedure for infectious and parasitic diseases

416 Septicemia, age > 17

418 Postoperative and posttraumatic infections

***Ventilation for Respiratory Disease (DRGs)***

475 Respiratory system diagnosis w ventilator support

***Tracheostomy (DRGs)***

541 Tracheostomy w mechanical vent 96+ hrs or principal dx except face, month, and neck diagnoses with major OR procedure.

542 Tracheostomy w mechanical vent 96+ hrs or principal dx except face, month, and neck diagnoses without major OR procedure.

**2006-2**

***Heart Attack (DRGs)***

121 Circulatory Disorders with Acute Myocardial Infarction and Major Complications, Discharged Alive

122 Circulatory Disorders with Acute Myocardial Infarction without Major Complications, Discharged Alive

123 Circulatory Disorders with Acute Myocardial Infarction, Expired

***Heart Failure (DRGs)***

127 Heart Failure and Shock

***Chest Pain (DRGs)***

143 Chest Pain

***Abnormal Heartbeat (DRGs)***

117 Cardiac Pacemaker Revision Except Device Replacement

118 Cardiac Pacemaker Device Replacement

124 Circulatory Disorders Except Acute Myocardial Infarction, with Cardiac Catheterization and Complex Diagnosis

125 Circulatory Disorders Except Acute Myocardial Infarction, with Cardiac Catheterization without Complex Diagnosis

138 Cardiac Arrhythmia and Conduction Disorders with CC

139 Cardiac Arrhythmia and Conduction Disorders without CC

551 Permanent Pacemaker Implant with MCV Diagnosis or AICD Lead or Generator

552 Other Permanent Pacemaker Implant without MCV Diagnosis

***Coronary Bypass (DRGs)***

103 Heart Transplant or Implant of Heart Assist System

106 Coronary Bypass with PTCA

108 Other Cardiothoracic Procedures

515 Cardiac Defibrillator Implant without Cardiac Catheterization

525 Heart Assist System Implant

535 Cardiac Defibrillator Implant with Cardiac Catheterization with Acute Myocardial Infarction, Heart Failure, or Shock

536 Cardiac Defibrillator Implant with Cardiac Catheterization without Acute Myocardial Infarction, Heart Failure, or Shock

547 Coronary Bypass with Cardiac Catheterization with MCV Diagnosis

548 Coronary Bypass with Cardiac Catheterization without MCV Diagnosis

549 Coronary Bypass without Cardiac Catheterization with MCV Diagnosis

550 Coronary Bypass without Cardiac Catheterization without MCV Diagnosis

***Heart Value Replacement (DRGs)***

104 Cardiac Valve Procedures and Other Major Cardiothoracic Procedures with Cardiac Catheterization

105 Cardiac Valve Procedures and Other Major Cardiothoracic Procedures without Cardiac Catheterization

***Percutaneous Transluminal Coronary Angioplasty (DRGs)***

518 Percutaneous Cardiovascular Procedures without Acute Myocardial Infarction without Coronary Artery Stent Implant

555 Percutaneous Cardiovascular Procedure with MCV Diagnosis

556 Percutaneous Cardiovascular Procedure with Non Drug-Eluting Stent without Major Cardiovascular Diagnosis

557 Percutaneous Cardiovascular Procedure with Drug-Eluting Stent with Major Cardiovascular Diagnosis

558 Percutaneous Cardiovascular Procedure with Drug-Eluting Stent without Major Cardiovascular Diagnosis

***Pneumonia (DRGs)***

079 Respiratory Infections and Inflammations, Age >17 with CC

080 Respiratory Infections and Inflammations, Age >17 without CC

089 Simple Pneumonia and Pleurisy, Age >17 with CC

090 Simple Pneumonia and Pleurisy, Age >17 without CC

***Asthma (DRGs)***

088 Chronic Obstructive Pulmonary Disease

096 Bronchitis and Asthma, Age >17 with CC

097 Bronchitis and Asthma, Age >17 without CC

098 Bronchitis and Asthma, Age 0-17

***Respiratory Failure (DRGs)***

087 Pulmonary Edema and Respiratory Failure

***Blood Clot in Lung (DRGs)***

078 Pulmonary Embolism

***Lung Repair (DRGs)***

075 Major Chest Procedures

076 Other Respiratory System O.R. Procedures with CC

077 Other Respiratory System O.R. Procedures without CC

***Hypotension (DRGs)***

141 Syncope and Collapse with CC

142 Syncope and Collapse without CC

***Blood Clot in Extremities (DRGs)***

128 Deep Vein Thrombophlebitis

129 Cardiac Arrest, Unexplained

130 Peripheral Vascular Disorders with CC

***Vascular Repair (DRGs)***

110 Major Cardiovascular Procedures with CC

111 Major Cardiovascular Procedures without CC

479 Other Vascular Procedures without CC

553 Other Vascular Procedures with CC with MCV Diagnosis

554 Other Vascular Procedures with CC without MCV Diagnosis

***Stroke (DRGs)***

014 Intracranial Hemorrhage or Cerebral Infarction

015 Nonspecific Cerebrovascular and Precerebral Occlusion without Infarction

559 Acute Ischemia Stroke with Use of Thrombolytic Agent

***Removal of Blockage in Head & Neck Vessel (DRGs)***

533 Extracranial Vascular Procedures with CC

534 Extracranial Vascular Procedures without CC

***Craniotomy (DRGs)***

001 Craniotomy, Age >17 with CC

002 Craniotomy, Age >17 without CC

528 Intracranial Vascular Procedures with Principal Diagnosis of Hemorrhage

529 Ventricular Shunt Procedures with CC

530 Ventricular Shunt Procedures without CC

543 Craniotomy with Implantation of Chemotherapeutic Agent or Acute Complex Central Nervous System Principal Diagnosis

***Diabetes (Principal Diagnosis ICD.9.CM codes)***

25000 DM2 UNCOMP NSU

25001 DM1 UNCOMP NSU

25002 DM2 UNCOMP UNC

25003 DM1 UNCOMP UNC

25010 DM2 W KETOACIDOSIS NSU

25011 DM1 W KETOACIDOSIS NSU

25012 DM2 W KETOACIDOSIS UNC

25013 DM1 W KETOACIDOSIS UNC

25020 DM2 W HYPEROSMOLARITY NSU

25021 DM1 W HYPEROSMOLARITY NSU

25022 DM2 W HYPEROSMOLARITY UNC

25023 DM1 W HYPEROSMOLARITY UNC

25030 DM2 W COMA NEC, NSU

25031 DM1 W COMA NEC, NSU

25032 DM2 W COMA NEC, UNC

25033 DM1 W COMA NEC, UNC

25040 DM2 W RENAL MANIFEST, NSU

25041 DM1 W RENAL MANIFEST, NSU

25042 DM2 W RENAL MANIFEST, UNC

25043 DM1 W RENAL MANIFEST, UNC

25050 DM2 W EYE MANIFEST, NSU

25051 DM1 W EYE MANIFEST, NSU

25052 DM2 W EYE MANIFEST, UNC

25053 DM1 W EYE MANIFEST, UNC

25060 DM2 W NEURO MANIFEST, NSU

25061 DM1 W NEURO MANIFEST, NSU

25062 DM2 W NEURO MANIFEST, UNC

25063 DM1 W NEURO MANIFEST, UNC

25070 DM2 W CIRC DISORD, NSU

25071 DM1 W CIRC DISORD, NSU

25072 DM2 W CIRC DISORD, UNC

25073 DM1 W CIRC DISORD, UNC

25080 DM2 W MANIFEST NEC, NSU

25081 DM1 W MANIFEST NEC, NSU

25082 DM2 W MANIFEST NEC, UNC

25083 DM1 W MANIFEST NEC, UNC

25090 DM2 Q COMP NOS, NSU

25091 DM1 Q COMP NOS, NSU

25092 DM2 Q COMP NOS, UNC

25093 DM1 Q COMP NOS, UNC

***Digestive Disease (DRGs)***

174 G.I. Hemorrhage with CC

175 G.I. Hemorrhage without CC

180 G.I. Obstruction with CC

181 G.I. Obstruction without CC

182 Esophagitis, Gastroenteritis and Miscellaneous Digestive Disorders, Age >17 with CC

183 Esophagitis, Gastroenteritis and Miscellaneous Digestive Disorders, Age >17 without CC

188 Other Digestive System Diagnoses, Age >17 with CC

189 Other Digestive System Diagnoses, Age >17 without CC

***Liver Disease (DRGs)***

202 Cirrhosis and Alcoholic Hepatitis

203 Malignancy of Hepatobiliary System or Pancreas

204 Disorders of Pancreas Except Malignancy

205 Disorders of Liver Except Malignancy, Cirrhosis and Alcoholic Hepatitis with CC

***Colorectal Repair (DRGs)***

146 Rectal Resection with CC

147 Rectal Resection without CC

148 Major Small and Large Bowel Procedures with CC

149 Major Small and Large Bowel Procedures without CC

***Gallbladder Removal (DRGs)***

195 Cholecystectomy with Common Duct Exploration with CC

196 Cholecystectomy with Common Duct Exploration without CC

197 Cholecystectomy Except By Laparoscope without Common Duct Exploration with CC

198 Cholecystectomy Except By Laparoscope without Common Duct Exploration without CC

493 Laparoscopic Cholecystectomy without Common Duct Exploration with CC

494 Laparoscopic Cholecystectomy without Common Duct Exploration without CC

***Stomach & Small Intestine Repair (DRGs)***

154 Stomach, Esophageal and Duodenal Procedures, Age >17 with CC

155 Stomach, Esophageal and Duodenal Procedures, Age >17 without CC

288 O.R. Procedures for Obesity

***Kidney Failure (DRGs)***

316 Renal Failure

***Kidney & Urinary Tract Infection (DRGs)***

321 Kidney and Urinary Tract Infections, Age >17 without CC

322 Kidney and Urinary Tract Infections, Age 0-17

***Prostatectomy (DRGs)***

306 Prostatectomy with CC

307 Prostatectomy without CC

334 Major Male Pelvic Procedures with CC

335 Major Male Pelvic Procedures without CC

336 Transurethral Prostatectomy with CC

337 Transurethral Prostatectomy without CC

***Medical Back (DRGs)***

243 Medical Back Problems

***Major Joint Repair (DRGs)***

210 Hip and Femur Procedures Except Major Joint Procedures, Age >17 with CC

211 Hip and Femur Procedures Except Major Joint Procedures, Age >17 without CC

471 Bilateral or Multiple Major Joint Procedures of Lower Extremity

545 Revision of Hip or Knee Replacement

***Neck/Back Repair (DRGs)***

496 Combined Anterior / Posterior Spinal Fusion

497 Spinal Fusion Except Cervical with CC

498 Spinal Fusion Except Cervical without CC

499 Back and Neck Procedures, Except Spinal Fusion, with CC

500 Back and Neck Procedures, Except Spinal Fusion, without CC

519 Cervical Spinal Fusion with CC

520 Cervical Spinal Fusion without CC

546 Spinal Fusions Except Cervical with Curvature of Spine or Malignancy

***Breast Cancer (DRGs)***

257 Total Mastectomy for Malignancy with CC

258 Total Mastectomy for Malignancy without CC

259 Subtotal Mastectomy for Malignancy with CC

260 Subtotal Mastectomy for Malignancy without CC

265 Skin Graft and/or Debridement Except for Skin Ulcer or Cellulitis with CC

266 Skin Graft and/or Debridement Except for Skin Ulcer or Cellulitis without CC

***Hysterectomy (DRGs)***

353 Pelvic Evisceration, Radical Hysterectomy and Radical Vulvectomy

354 Uterine and Adnexa Procedures for Nonovarian/Adnexal Malignancy with CC

355 Uterine and Adnexa Procedures for Nonovarian/Adnexal Malignancy without CC

357 Uterine and Adnexa Procedures for Ovarian or Adnexal Malignancy

358 Uterine and Adnexa Procedures for Nonmalignancy with CC

359 Uterine and Adnexa Procedures for Nonmalignancy without CC

***Infectious Disease (DRGs)***

415 O.R. Procedure for Infectious and Parasitic Diseases

416 Septicemia, Age > 17

418 Postoperative and Posttraumatic Infections

***Ventilation for Respiratory Disease (DRGs)***

475 Respiratory System Diagnosis with Ventilator Support

***Tracheostomy (DRGs)***

541 ECMO or Tracheostomy with Mechanical Ventilation 96+ Hours or Principal Diagnosis Except Face, Mouth, and Neck with Major O.R.

542 Tracheostomy with Mechanical Ventilation 96+ Hours or Principal Diagnosis Except Face, Mouth, and Neck without Major O.R.

**2007-4**

***Heart Attack ( MS-DRGs)***

280 Acute myocardial infarction, discharged alive with MCC

281 Acute myocardial infarction, discharged alive with CC

282 Acute myocardial infarction, discharged alive without CC/MCC

283 Acute myocardial infarction, expired with MCC

284 Acute myocardial infarction, expired with CC

285 Acute myocardial infarction, expired without CC/MCC

***Heart Failure (MS-DRGs)***

291 Heart failure and shock with MCC

292 Heart failure and shock with CC

293 Heart failure and shock without CC/MCC

***Chest Pain (MS-DRGs)***

313 Chest pain

***Abnormal Heartbeat (MS-DRGs)***

242 Permanent cardiac pacemaker implant with MCC

243 Permanent cardiac pacemaker implant with CC

244 Permanent cardiac pacemaker implant without CC/MCC

245 AICD lead and generator procedures

258 Cardiac pacemaker device replacement with MCC

259 Cardiac pacemaker device replacement without MCC

260 Cardiac pacemaker revision except device replacement with MCC

261 Cardiac pacemaker revision except device replacement with CC

262 Cardiac pacemaker revision except device replacement without CC/MCC

286 Circulatory disorders except AMI with cardiac cath with MCC

287 Circulatory disorders except AMI with cardiac cath without MCC

308 Cardiac arrhythmia and conduction disorders with MCC

309 Cardiac arrhythmia and conduction disorders with CC

310 Cardiac arrhythmia and conduction disorders without CC/MCC

***Coronary Bypass (MS-DRGs)***

001 Heart transplant or implant of heart assist system with MCC

002 Heart transplant or implant of heart assist system without MCC

215 Other heart assist system implant

222 Cardiac defibrillator implant with cardiac cath with AMI, heart failure or shock with MCC

223 Cardiac defibrillator implant with cardiac cath with AMI, heart failure or shock without MCC

224 Cardiac defibrillator implant with cardiac cath without AMI, heart failure or shock with MCC

225 Cardiac defibrillator implant with cardiac cath without AMI, heart failure or shock without MCC

226 Cardiac defibrillator implant without cardiac cath with MCC

227 Cardiac defibrillator implant without cardiac cath without MCC

228 Other cardiothoracic procedures with MCC

229 Other cardiothoracic procedures with CC

230 Other cardiothoracic procedures without CC/MCC

231 Coronary bypass with PTCA with MCC

232 Coronary bypass with PTCA without MCC

233 Coronary bypass with cardiac cath with MCC

234 Coronary bypass with cardiac cath without MCC

235 Coronary bypass without cardiac cath with MCC

236 Coronary bypass without cardiac cath without MCC

***Heart Value Replacement (MS-DRGs)***

216 Cardiac valve and other major cardiothoracic procedure with cardiac cath with MCC

217 Cardiac valve and other major cardiothoracic procedure with cardiac cath with CC

218 Cardiac valve and other major cardiothoracic procedure with cardiac cath without CC/MCC

219 Cardiac valve and other major cardiothoracic procedure without cardiac cath with MCC

220 Cardiac valve and other major cardiothoracic procedure without cardiac cath with CC

221 Cardiac valve and other major cardiothoracic procedure without cardiac cath without CC/MCC

***Percutaneous Transluminal Coronary Angioplasty (MS-DRGs)***

246 Percutaneous cardiovascular procedure with drug-eluting stent with MCC or 4 or more

vessels/stents

247 Percutaneous cardiovascular procedure with drug-eluting stent without MCC

248 Percutaneous cardiovascular procedure with non-drug-eluting stent with MCC or 4 or more vessels/stents

249 Percutaneous cardiovascular procedure with non-drug-eluting stent without MCC

250 Percutaneous cardiovascular procedure without coronary artery stent or AMI with MCC

251 Percutaneous cardiovascular procedure without coronary artery stent or AMI without

MCC

***Pneumonia (MS-DRGs)***

177 Respiratory infections and inflammations with MCC

178 Respiratory infections and inflammations with CC

179 Respiratory infections and inflammations without CC/MCC

193 Simple pneumonia and pleurisy with MCC

194 Simple pneumonia and pleurisy with CC

195 Simple pneumonia and pleurisy without CC/MCC

***Asthma (MS-DRGs)***

190 Chronic obstructive pulmonary disease with MCC

191 Chronic obstructive pulmonary disease with CC

192 Chronic obstructive pulmonary disease without CC/MCC

202 Bronchitis and asthma with CC/MCC

203 Bronchitis and asthma without CC/MCC

***Respiratory Failure (MS-DRGs)***

189 Pulmonary edema and respiratory failure

***Blood Clot in Lung (MS-DRGs)***

175 Pulmonary embolism with MCC

176 Pulmonary embolism without MCC

***Lung Repair (MS-DRGs)***

163 Major chest procedures with MCC

164 Major chest procedures with CC

165 Major chest procedures without CC/MCC

166 Other respiratory system O.R. procedures with MCC

167 Other respiratory system O.R. procedures with CC

168 Other respiratory system O.R. procedures without CC/MCC

***Hypotension (MS-DRGs)***

312 Syncope and collapse

***Blood Clot in Extremities (MS-DRGs)***

294 Deep vein thrombophlebitis with CC/MCC

295 Deep vein thrombophlebitis without CC/MCC

299 Peripheral vascular disorders with MCC

300 Peripheral vascular disorders with CC

301 Peripheral vascular disorders without CC/MCC

***Vascular Repair (MS-DRGs)***

237 Major cardiovascular procedures with MCC or thoracic aortic aneurysm repair

238 Major cardiovascular procedures without MCC

252 Other vascular procedures with MCC

253 Other vascular procedures with CC

254 Other vascular procedures without CC/MCC

***Stroke (MS-DRGs)***

061 Acute ischemic stroke with use of thrombolytic agent with MCC

062 Acute ischemic stroke with use of thrombolytic agent with CC

063 Acute ischemic stroke with use of thrombolytic agent without CC/MCC

064 Intracranial hemorrhage or cerebral infarction with MCC

065 Intracranial hemorrhage or cerebral infarction with CC

066 Intracranial hemorrhage or cerebral infarction without CC/MCC

067 Nonspecified CVA and precerebral occlusion without infarct with MCC

068 Nonspecified CVA and precerebral occlusion without infarct without MCC

***Removal of Blockage in Head & Neck Vessel (MS-DRGs)***

034 Carotid artery stent procedure with MCC

035 Carotid artery stent procedure with CC

036 Carotid artery stent procedure without CC/MCC

037 Extracranial procedures with MCC

038 Extracranial procedures with CC

039 Extracranial procedures without CC/MCC

***Craniotomy (MS-DRGs)***

020 Intracranial vascular procedures with PDX of hemorrhage with MCC

021 Intracranial vascular procedures with PDX of hemorrhage with CC

022 Intracranial vascular procedures with PDX of hemorrhage without CC/MCC

023 Craniotomy with major device implant or acute complex CNS PDX with MCC or chemo implant

024 Craniotomy with major device implant or acute complex CNS PDX without MCC

025 Craniotomy and endovascular intracranial procedures with MCC

026 Craniotomy and endovascular intracranial procedures with CC

027 Craniotomy and endovascular intracranial procedures without CC/MCC

031 Ventricular shunt procedures with MCC

032 Ventricular shunt procedures with CC

033 Ventricular shunt procedures without CC/MCC

***Diabetes (Principal Diagnosis ICD.9.CM codes)***

25000 Diabetes mellitus without mention of complication, Type II or unspecified type, not

stated as uncontrolled

25001 Diabetes mellitus without mention of complication, Type I [juvenile type], not stated as Uncontrolled

25002 Diabetes mellitus without mention of complication, Type II or unspecified type, uncontrolled

25003 Diabetes mellitus without mention of complication, Type I [juvenile type], uncontrolled

25010 Diabetes with ketoacidosis, Type II or unspecified type, not stated as uncontrolled

25011 Diabetes with ketoacidosis, Type I [juvenile type], not stated as uncontrolled

25012 Diabetes with ketoacidosis, Type II or unspecified type, uncontrolled

25013 Diabetes with ketoacidosis, Type I [juvenile type], uncontrolled

25020 Diabetes with hyperosmolarity, Type II or unspecified type, not stated as uncontrolled

25021 Diabetes with hyperosmolarity, Type I [juvenile type], not stated as uncontrolled

25022 Diabetes with hyperosmolarity, Type II or unspecified type, uncontrolled

25023 Diabetes with hyperosmolarity, Type I [juvenile type], uncontrolled

25030 Diabetes with other coma, Type II or unspecified type, not stated as uncontrolled

25031 Diabetes with other coma, Type I [juvenile type], not stated as uncontrolled

25032 Diabetes with other coma, Type II or unspecified type, uncontrolled

25033 Diabetes with other coma, Type I [juvenile type], uncontrolled

25040 Diabetes with renal manifestations, Type II or unspecified type, not stated as uncontrolled

25041 Diabetes with renal manifestations, Type I [juvenile type], not stated as uncontrolled

25042 Diabetes with renal manifestations, Type II or unspecified type, uncontrolled

25043 Diabetes with renal manifestations, Type I [juvenile type], uncontrolled

25050 Diabetes with ophthalmic manifestations, Type II or unspecified type, not stated as uncontrolled

25051 Diabetes with ophthalmic manifestations, Type I [juvenile type], not stated as uncontrolled

25052 Diabetes with ophthalmic manifestations, Type II or unspecified type, uncontrolled

25053 Diabetes with ophthalmic manifestations, Type I [juvenile type], uncontrolled

25060 Diabetes with neurological manifestations, Type II or unspecified type, not stated as uncontrolled

25061 Diabetes with neurological manifestations, Type I [juvenile type], not stated as uncontrolled

25062 Diabetes with neurological manifestations, Type II or unspecified type, uncontrolled

25063 Diabetes with neurological manifestations, Type I [juvenile type], uncontrolled

25070 Diabetes with peripheral circulatory disorders, Type II or unspecified type, not stated as uncontrolled

25071 Diabetes with peripheral circulatory disorders, Type I [juvenile type], not stated as uncontrolled

25072 Diabetes with peripheral circulatory disorders, Type II or unspecified type, uncontrolled

25073 Diabetes with peripheral circulatory disorders, Type I [juvenile type], uncontrolled

25080 Diabetes with other specified manifestations, Type II or unspecified type, not stated as uncontrolled

25081 Diabetes with other specified manifestations, Type I [juvenile type], not stated as uncontrolled

25082 Diabetes with other specified manifestations, Type II or unspecified type, uncontrolled

25083 Diabetes with other specified manifestations, Type I [juvenile type], uncontrolled

25090 Diabetes with unspecified complication, Type II or unspecified type, not stated as uncontrolled

25091 Diabetes with unspecified complication, Type I [juvenile type], not stated as uncontrolled

25092 Diabetes with unspecified complication, Type II or unspecified type, uncontrolled

25093 Diabetes with unspecified complication, Type I [juvenile type], uncontrolled

***Digestive Disease (MS-DRGs)***

368 Major esophageal disorders with MCC

369 Major esophageal disorders with CC

370 Major esophageal disorders without CC/MCC

371 Major gastrointestinal disorders and peritoneal infections with MCC

372 Major gastrointestinal disorders and peritoneal infections with CC

373 Major gastrointestinal disorders and peritoneal infections without CC/MCC

377 GI hemorrhage with MCC

378 GI hemorrhage with CC

379 GI hemorrhage without CC/MCC

388 GI obstruction with MCC

389 GI obstruction with CC

390 GI obstruction without CC/MCC

391 Esophagitis, gastroenteritis and misc digestive disorders with MCC

392 Esophagitis, gastroenteritis and misc digestive disorders without MCC

393 Other digestive system diagnoses with MCC

394 Other digestive system diagnoses with CC

395 Other digestive system diagnoses without CC/MCC

***Liver Disease (MS-DRGs)***

432 Cirrhosis and alcoholic hepatitis with MCC

433 Cirrhosis and alcoholic hepatitis with CC

434 Cirrhosis and alcoholic hepatitis without CC/MCC

435 Malignancy of hepatobiliary system or pancreas with MCC

436 Malignancy of hepatobiliary system or pancreas with CC

437 Malignancy of hepatobiliary system or pancreas without CC/MCC

438 Disorders of pancreas except malignancy with MCC

439 Disorders of pancreas except malignancy with CC

440 Disorders of pancreas except malignancy without CC/MCC

441 Disorders of liver except malignancy, cirrhosis, alcoholic hepatitis with MCC

442 Disorders of liver except malignancy, cirrhosis, alcoholic hepatitis with CC

443 Disorders of liver except malignancy, cirrhosis, alcoholic hepatitis without CC/MCC

***Colorectal Repair (MS-DRGs)***

329 Major small and large bowel procedures with MCC

330 Major small and large bowel procedures with CC

331 Major small and large bowel procedures without CC/MCC

332 Rectal resection with MCC

333 Rectal resection with CC

334 Rectal resection without CC/MCC

***Gallbladder Removal (MS-DRGs)***

411 Cholecystectomy with common duct exploration with MCC

412 Cholecystectomy with common duct exploration with CC

413 Cholecystectomy with common duct exploration without CC/MCC

414 Cholecystectomy except by laparoscope without common duct exploration with MCC

415 Cholecystectomy except by laparoscope without common duct exploration with CC

416 Cholecystectomy except by laparoscope without common duct exploration without CC/MCC

417 Laparoscopic cholecystectomy without common duct exploration with MCC

418 Laparoscopic cholecystectomy without common duct exploration with CC

419 Laparoscopic cholecystectomy without common duct exploration without CC/MCC

***Stomach & Small Intestine Repair (MS-DRGs)***

326 Stomach, esophageal and duodenal procedures with MCC

327 Stomach, esophageal and duodenal procedures with CC

328 Stomach, esophageal and duodenal procedures without CC/MCC

619 O.R. procedures for obesity with MCC

620 O.R. procedures for obesity with CC

621 O.R. procedures for obesity without CC/MCC

***Kidney Failure (MS-DRGs)***

682 Renal failure with MCC

683 Renal failure with CC

684 Renal failure without CC/MCC

***Kidney & Urinary Tract Infection (MS-DRGs)***

689 Kidney and urinary tract infections with MCC

690 Kidney and urinary tract infections without MCC

***Prostatectomy (MS-DRGs)***

665 Prostatectomy with MCC

666 Prostatectomy with CC

667 Prostatectomy without CC/MCC

707 Major male pelvic procedures with CC/MCC

708 Major male pelvic procedures without CC/MCC

713 Transurethral prostatectomy with CC/MCC

714 Transurethral prostatectomy without CC/MCC

***Medical Back (MS-DRGs)***

551 Medical back problems with MCC

552 Medical back problems without MCC

***Major Joint Repair (MS-DRGs)***

461 Bilateral or multiple major joint procedures of lower extremity with MCC

462 Bilateral or multiple major joint procedures of lower extremity without MCC

466 Revision of hip or knee replacement with MCC

467 Revision of hip or knee replacement with CC

468 Revision of hip or knee replacement without CC/MCC

469 Major joint replacement or reattachment of lower extremity with MCC

470 Major joint replacement or reattachment of lower extremity without MCC

480 Hip and femur procedures except major joint with MCC

481 Hip and femur procedures except major joint with CC

482 Hip and femur procedures except major joint without CC/MCC

***Neck/Back Repair (MS-DRGs)***

453 Combined anterior/posterior spinal fusion with MCC

454 Combined anterior/posterior spinal fusion with CC

455 Combined anterior/posterior spinal fusion without CC/MCC

456 Spinal fusion except cervical with spinal curvature, malignancy, infection or 9 or more fusions with MCC

457 Spinal fusion except cervical with spinal curvature, malignancy, infection or 9 or more fusions with CC

458 Spinal fusion except cervical with spinal curvature, malignancy, infection or 9 or more fusions without CC/MCC

459 Spinal fusion except cervical with MCC

460 Spinal fusion except cervical without MCC

471 Cervical spinal fusion with MCC

472 Cervical spinal fusion with CC

473 Cervical spinal fusion without CC/MCC

490 Back and neck procedure except spinal fusion with CC/MCC or disc device/neurostimulator

491 Back and neck procedure except spinal fusion without CC/MCC

***Breast Cancer (MS-DRGs)***

576 Skin graft and/or debridement except for skin ulcer or cellulitis with MCC

577 Skin graft and/or debridement except for skin ulcer or cellulitis with CC

578 Skin graft and/or debridement except for skin ulcer or cellulitis without CC/MCC

582 Mastectomy for malignancy with CC/MCC

583 Mastectomy for malignancy without CC/MCC

584 Breast biopsy, local excision and other breast procedures with CC/MCC

585 Breast biopsy, local excision and other breast procedures without CC/MCC

***Hysterectomy (MS-DRGs)***

734 Pelvic evisceration, radical hysterectomy and radical vulvectomy with CC/MCC

735 Pelvic evisceration, radical hysterectomy and radical vulvectomy without CC/MCC

736 Uterine and adnexa procedure for ovarian or adnexal malignancy with MCC

737 Uterine and adnexa procedure for ovarian or adnexal malignancy with CC

738 Uterine and adnexa procedure for ovarian or adnexal malignancy without CC/MCC

739 Uterine and adnexa procedure for non

740 Uterine and adnexa procedure for non

741 Uterine and adnexa procedure for non

742 Uterine and adnexa procedure for non

743 Uterine and adnexa procedure for non

***Infectious Disease (MS-DRGs)***

853 Infectious and parasitic diseases with O.R. procedure with MCC

854 Infectious and parasitic diseases with O.R. procedure with CC

855 Infectious and parasitic diseases with O.R. procedure without CC/MCC

856 Postoperative or post-traumatic infections with O.R. procedure with MCC

857 Postoperative or post-traumatic infections with O.R. procedure with CC

858 Postoperative or post-traumatic infections with O.R. procedure without CC/MCC

862 Postoperative and post-traumatic infections with MCC

863 Postoperative and post-traumatic infections without MCC

870 Septicemia with mechanical ventilation 96+ hrs

871 Septicemia without mechanical ventilation 96+ hrs with MCC

872 Septicemia without mechanical ventilation 96+ hrs without MCC

***Ventilation for Respiratory Disease (MS-DRGs)***

207 Respiratory system diagnosis with ventilator support 96+ hrs.

208 Respiratory system diagnosis with ventilator support <96 hrs.

***Tracheostomy (MS-DRGs)***

003 ECMO or tracheostomy with mechanical ventilation 96+ hrs or PDX except face, mouth, and neck with major O.R. procedure

004 Tracheostomy with mechanical ventilation 96+ hrs or PDX except face, mouth, and neck without major O.R. procedure
